# The Biology of SUMO-Targeted Ubiquitin Ligases in Drosophila Development, Immunity, and Cancer

**DOI:** 10.3390/jdb6010002

**Published:** 2018-01-01

**Authors:** Mona Abed, Eliya Bitman-Lotan, Amir Orian

**Affiliations:** 1 Genentech, 1 DNA Way, South San Francisco, CA 94080, USA; abed.monah@gene.com; 2 Rappaport Research Institute and Faculty of Medicine, Technion-Israel Institute of Technology, Haifa 3109610, Israel; eliyabit@technion.ac.il

**Keywords:** ubiquitin, SUMO, Dgrn, SUMO-targeted ubiquitin ligases RNF4, transcription, gene regulation, development, immunity, cancer

## Abstract

The ubiquitin and SUMO (small ubiquitin-like modifier) pathways modify proteins that in turn regulate diverse cellular processes, embryonic development, and adult tissue physiology. These pathways were originally discovered biochemically in vitro, leading to a long-standing challenge of elucidating both the molecular cross-talk between these pathways and their biological importance. Recent discoveries in *Drosophila* established that ubiquitin and SUMO pathways are interconnected via evolutionally conserved SUMO-targeted ubiquitin ligase (STUbL) proteins. STUbL are RING ubiquitin ligases that recognize SUMOylated substrates and catalyze their ubiquitination, and include Degringolade (Dgrn) in *Drosophila* and RNF4 and RNF111 in humans. STUbL are essential for early development of both the fly and mouse embryos. In the fly embryo, Dgrn regulates early cell cycle progression, sex determination, zygotic gene transcription, segmentation, and neurogenesis, among other processes. In the fly adult, Dgrn is required for systemic immune response to pathogens and intestinal stem cell regeneration upon infection. These functions of Dgrn are highly conserved in humans, where RNF4-dependent ubiquitination potentiates key oncoproteins, thereby accelerating tumorigenesis. Here, we review the lessons learned to date in *Drosophila* and highlight their relevance to cancer biology.

## 1. Ubiquitination, SUMOylation, and Their Enzymatic Connectors

Post-translational modifications (PTMs) are the regulatory, reversible covalent linkage of small chemical groups or proteins to existing proteins during or after translation. 

The discovery 30 years ago of ubiquitination, i.e., tagging a protein with ubiquitin, opened the door to a new area of post-transcriptional modification [1]. Ubiquitination involves the enzymatic covalent attachment of a ubiquitin molecule (Ub) to a substrate protein as a monomer (mono-ubiquitination), or to an existing Ub moiety already attached to the substrate, thus generating a multi-Ub chain (poly-ubiquitination). This process is highly regulated and is mediated by three enzymes: E1 (ubiquitin-activating enzyme), E2 (ubiquitin-conjugating enzyme, Ubc), and E3 (ubiquitin-protein ligase). The latter interacts directly with the substrate and harbors a substrate recognition region/motif [2]. Ubiquitination can take place on Lys residues, the free N-termini of proteins, and was recently shown to occur also on Ser residues [3]. Each ubiquitin molecule contains seven internal lysine residues, each of which can serve as a target for conjugation of additional ubiquitin molecules. These ubiquitin chains can be either homotypic (i.e., contain a single type of internal link) or heterotypic (contain diverse internal links). This internal Ub linkage determines the structure of the ubiquitin chain, and linkage-specific processes are currently emerging [4]. For example, homotypic K48-polyUb chains target proteins for degradation by 26S proteasomes, a nano-machine complex that is proficient in the proteolytic degradation of ubiquitinated proteins [5]. Ubiquitination may be reversed by deubiquitinating enzymes (DUBs) that catalyze the hydrolysis of the iso-peptide bond, thus removing the ubiquitin moiety from the protein [4]. As such, the ubiquitin system regulates diverse cellular and developmental processes from receptors endocytosis at the cytoplasmic membrane to the regulation of gene expression in the nucleus. Therefore, perturbations in the ubiquitin pathway are tightly associated with pathological conditions such as congenital and developmental syndromes, cancer, and neurodegeneration [6].

Several ubiquitin-like molecules and pathways (UbLs) have been discovered in recent decades [7]. The conjugation of each of these UbLs to a targeted protein requires ATP-dependent activation of the UbL and the attachment activity of a UbL-specific enzymatic machinery. To date, the best-characterized UbL is the small ubiquitin-like modifier (SUMO). SUMOylation is highly analogous to ubiquitination in terms of the enzyme cascade (E1, E2, and E3-SUMO ligase enzymes) that covalently adds a single SUMO protein or poly-SUMO-chain to the substrate protein. Similar to ubiquitination, SUMOylation is also reversible and mediated by SUMO-specific peptidases (Ulps/SENPs) [8,9,10]. SUMOylation plays a critical role in *Drosophila* development. Genetic and biochemical characterization of SUMOylation in the *Drosophila* melanogaster revealed many of its conserved biological functions, which include regulation of key signaling pathways, developmentally regulated transcription factors, as well as the response to DNA damage and DNA replication [11]. 

Both ubiquitin and SUMO regulate protein-protein interactions as well as protein stability, function, and localization. Moreover, substrates can be modified by both PTMs in a step-wise manner that includes the potential generation of heterotypic ubiquitin-SUMO chains [12]. As outlined in this short review, SUMO-targeted ubiquitin ligase (STUbL) is a family of RING ubiquitin ligases that connect the ubiquitin and SUMO pathways. Here, we discuss their function in the context of *Drosophila* development and physiology, and the relevance of lessons gained in the fly system to cancer biology [13,14].

**Figure 1:**
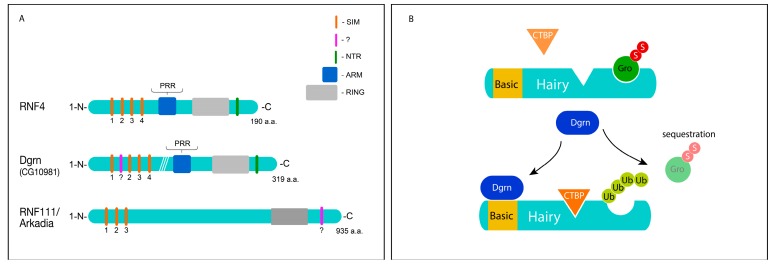
Structure of STUbL protein and a putative role for Dgrn in the regulation of co-factors selection. (**A**) Schematic diagram of human and *Drosophila* STUbL proteins (not to scale). SIM, SUMO-interacting motif; NTR, nucleosome-targeting region; ARM, arginine-rich region, and PRR are highly conserved amino acids within the ARM; RING, Really Interesting New Gene catalytic domain. “?” denotes putative domains. (**B**) A model for the Dgrn-dependent inactivation of Hairy-Gro interaction, see text for details.

STUbLs are E3 ubiquitin ligases that bind non-covalently to the SUMO moiety of SUMOylated proteins via their SUMO-interacting motifs (SIMs) (Figure 1A). Once bound, STUbLs mediate the ubiquitination of the SUMOylated protein, in many cases targeting it for proteasomal degradation [14]. The vertebrate genome codes for two STUbL genes, RNF4 and Arkedia (RNF111) [14,15]. The *Drosophila* genome encodes for a single STUbL protein, which is highly similar to both mammalian proteins termed Degringolade (Dgrn, CG10981) [16]. Analysis of Dgrn in flies showed that it plays key roles in early embryonic development [17], DNA repair [18], and regulation of the immune response [19]. 

## 2. Dgrn, Early *Drosophila* Embryogenesis, and DNA Repair

The early *Drosophila* embryo develops as a closed system orchestrated mainly by maternally contributed mRNAs and proteins. Fertilization triggers rapid nuclear divisions without accompanying cytokinesis. At nuclear cycles 8–10, the majority of nuclei migrate to right under the plasma membrane at the surface of the embryo, forming the syncytial blastoderm. At this stage of embryogenesis, these cells will undergo additional rapid nuclear divisions with neighboring mitotic spindles originating from distinctive centromere pairs. Finally, at cycle 14, the embryo undergoes cellularization. Post-translational modifications have been shown to regulate these early cycles, in agreement with the lack of active transcription [20].

Dgrn, which is maternally contributed and ubiquitously distributed throughout the embryo, plays an important role in these stages of early embryogenesis. Indeed, while homozygote *dgrn* null mutant flies develop to adulthood, homozygous females are sterile, laying embryos that do not hatch. The majority of these embryos arrest during early nuclear cycles 2–3 [16]. A small portion of them arrest several cycles later, yet are unable to anchor their nuclei to the centromeres at the embryo periphery. Phenotypically, this results in nuclei that “fall” from the periphery to the center of the embryo, leaving behind cells with empty centromeres that resemble halos and atypical mitotic chromatin structures [16].

Analysis of *dgrn* mutants unveiled that these nuclei as well as the entire embryo are enriched for SUMOylated proteins, further exemplifying the role of Dgrn in regulating SUMOylation [17]. These findings fit well with the observation that SUMOylated proteins are required for maintaining DNA integrity during early development. For example, *Smt3* (SUMO)-mutant embryos have been shown to display early nuclear cycle defects including irregular size and distribution of nuclei [21]. Moreover, DNA repair and heterochromatin proteins that are conserved from flies to humans, such as Blm, WRN, Rad52, RPA, RAP80, PCNA, RecQ, are regulated by SUMOylation [22,23,24,25]. Indeed, STUbLs are key players in resolving double-strand breaks (DSBs) in heterochromatin in yeast, flies, and humans [18,24,25,26,27,28]. Yeast deficient in Dgrn ortholog *Slx5-Slx8* genes accumulate DNA lesions during replication [29]. Of specific interest are repetitive sequences (repeats) within heterochromatin that challenge genome stability maintenance, given the potential ability of repeats to recombine with similar sequences within the genome. Specific mechanisms therefore evolved to prevent aberrant recombination. Ryu et al. [18] showed that SUMOylation of repair proteins prevents recruitment of the DSB repair protein Rad51 when the DSB is localized in the heterochromatin. Such DSBs are, however, repaired at the nuclear periphery adjunct to nuclear pores. Dgrn likely removes the Rad51 block by ubiquitination and subsequent degradation of the SUMOylated proteins, preventing DSB repair at the heterochromatin and enabling DSB repair to progress. In mouse and human cells, RNF4 is similarly required for DSB repair and for the response to DNA damage. RNF4 substrates include essential mediators of the DNA damage response such as RAP80 [25], Fanconi anemia ID protein complex [30,31], and MDC1 [28,32]. Thus, supporting the notion that a STUbL-dependent mechanism connects SUMOylation and ubiquitination at DNA damage sites. Indeed, RNF4-hypomorphic mouse mutants exhibit hypersensitivity to genotoxic stress and ionizing radiation in vivo [27].

## 3. The Role of Dgrn in Transcriptional Repression

STUbLs recognize SUMOylated proteins and catalyze their ubiquitination, which in many cases results in proteasomal degradation. For example, the mammalian STUbL RNF4 ubiquitinates the SUMOylated Promyelocytic leukemia protein (PML) and its oncogenic fusion PML-RAR. This SUMO-mediated and RNF4-dependent ubiquitination subsequently leads to proteasomal degradation of the PML or PML-RAR proteins. In this context, RNF4 inhibits tumorigenesis and its expression in PML leukemic cells results in their differentiation [33]. Numerous proteins bound by the SUMO interacting motifs (SIMs) of RNF4 were likewise identified [34]. However, STUbLs also have an SUMO-independent mode of substrate recognition and were shown not only to target proteins for degradation, but also to regulate protein-protein interactions and protein stability (Figure 1B) [13,17,35]. 

A well-studied case in this context is the role of Dgrn in transcriptional repression during *Drosophila* development [17]. Dgrn was initially identified in a two-hybrid screen of the basic helix-loop-helix (bHLH) repressor Hairy [36], in which the interaction between Dgrn and Hairy was found to be independent of SUMOylation and was mediated by the RING domain of Dgrn and the basic region of Hairy. Given the conservation of the basic domain among all Hairy/Enhancer of Split/Deadpan (HES) family repressors, Dgrn interacted physically with the entire HES family of proteins, assembling heterotypic poly-ubiquitin chains on these repressors, with the exception of Her, which differs in its basic region and is not bound and ubiquitinated by Dgrn. Moreover, observations in yeast and vertebrates suggest that other STUbL proteins also bind and ubiquitinate substrates in a SUMO-independent manner. For example, the yeast STUbL SLx5-Slx8 was shown to recognize the MATα repressor independent of SUMOylation [37].

Dgrn-mediated ubiquitination of HES proteins did not lead to their degradation but rather selectively affected their ability to recruit co-repressors and regulate their function. For example, the Hairy repressor functions by recruiting co-repressors such as Groucho (Gro)/TLE, dSir2, or dCtBP [38,39]. Together with its co-repressors, Hairy regulates segmentation and neurogenesis. However, the selection mechanism that determines the association of Hairy with an individual co-repressor is not well understood. In this regard, Dgrn-mediated ubiquitination selectively reduces the affinity of Hairy to its co-repressor (Gro) but does not impact its ability to interact with its other co-factors such as dCtBP. These co-factors bind to different sites within Hairy, and Dgrn-mediated catalysis of heterotypic poly-ubiquitin chains on Hairy may mask specific co-factor binding sites (such as the WPRW Gro-binding site), but not others sites required for binding of the other cofactors (Figure 1B).

The action of Dgrn is also aimed at the co-repressor Gro/TLE. SUMOylation of the Gro/TLE co-repressor complex modulates its co-repressor activity [40]. At the same time, however, SUMOylation of Gro contains a self-limiting mechanism. Biochemical and immuno-histological analyses suggest that Dgrn targets SUMOylated-Gro for sequestration (rather than degradation), leading to inactivation of the co-repressor (Figure 1B), a mechanism that is likely reversible. DUBs and SENPs that reverse the ubiquitination of Hairy as well as Gro SUMOylation by removing the ubiquitin and SUMO chains, respectively, are potentially capable of restoring Hairy~Gro repressive activity. This model of Dgrn-dependent sequestration and recycling is attractive, as Gro is a stable protein, which is used numerous times during development and in adult tissues. Indeed, Dgrn was able to alleviate Gro-dependent repression in biological contexts other than Hairy-regulated processes [16,17]. 

It should, however, be noted that, unlike the established interaction between Hairy and Dgrn, there is no evidence of direct binding between endogenous SUMOylated-Gro and Dgrn. Thus, it is reasonable also to consider an indirect effect of Dgrn on the SUMOylation machinery with similar outcomes. This possibility is supported by a recent observation that RNF4 targets multiple enzymes within the SUMO conjugation machinery for degradation, such as the SUMO E2 enzyme Ubc9, several SUMO E3 ligases PIAS1, PIAS2, PIAS3, ZNF451, and NSMCE2 [41].

**Figure 2:**
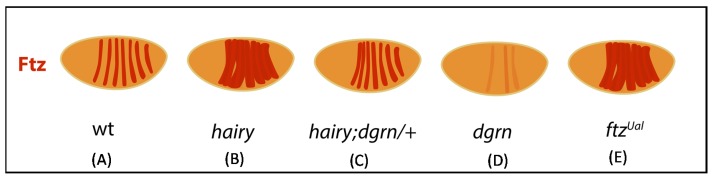
Post-transcriptional regulation of Ftz during segmentation. Cartoon depicting the pattern of Ftz protein during segmentation. (**A**) Endogenous Ftz protein expression is limited to seven stripes. (**B**) Ftz protein expression is extended in *hairy* mutants. (**C**) Ftz expression in *hairy* mutant embryos that are also heterozygous for *dgrn^DK^* (*hairy*; *dgrn^DK^*/*+*) is greatly restored [17]. (**D**) Ftz protein expression is reduced in Dgrn-null embryos [16]. (**E**) Ftz protein expression is expanded in *ftz^Ual^* embryos harboring mutations in a short “stability” motif that is required for Ftz degradation [44].

Biologically, the finding that Dgrn reduces binding between Hairy and Gro is manifested when Hairy-dependent repression is observed during embryogenesis. Hairy functions in the developing *Drosophila* embryo as a primary pair-rule gene that establishes reiterative patterning by repressing the expression of *fushi-tarazu* (*ftz*) [42]. Hairy-hypomorphic embryos therefore display aberrant segmentation and expansion of *ftz* expression, including at protein level. This phenotype is suppressed when *dgrn* levels are co-reduced (mutants of *hairy* that are also heterozygous for *dgrn*). Moreover, Ftz protein level is reduced in *dgrn*-null embryos [16], which might result from an increase in Hairy-Gro mediated repression of Ftz mRNA transcription in the absence of Dgrn. It may also, however, reflect the direct impact of Dgrn on Ftz protein stability as speculated below (Figure 2). 

While extensive research focused on the transcriptional control of segmentation, less is known regarding the contribution of protein stability and degradation to *Drosophila* segmentation. For example, expression of Ftz under regulation of *hb* promotor resulted in Ftz protein expression that initially recapitulated the *hb* pattern of expression at protein level. Over time, however, this *hb* pattern dissolved and was replaced with the classical striped pattern of the endogenous Ftz protein [43]. In addition, a short, 12 amino acid long stability motif within Ftz protein controls Ftz protein stability, and *ftz-ultra abdominal* (*ftz^UAl^*) mutants carrying mutations in a critical Pro residues within this motif exhibit expanded Ftz protein expression (Figure 2) [44]. 

Moreover, the Ftz stability motif is highly similar to the stability motif present in c-Myc, which regulates c-Myc degradation and stabilization by RNF4 (see Section 6) [35,45]. Interestingly, the stability motif includes a conserved stabilizing Ser residue (Ser62 in c-Myc) that is critical for recognition by RNF4 and is present in Ftz, Hb, Prd, and Eve. Thus, it seems that ubiquitin-mediated degradation, together with RNF4-dependent protein stabilization, may be an additional level of regulation that shapes segmentation at the post-transcriptional level. 

A second phenotype associated with Hairy is the appearance of ectopic bristles that are observed on the wing margin of *hairy*^1^ hypomorphic mutant adults [46]. These phenotypes are also suppressed by Dgrn heterozygosity. Moreover, in gain-of-function experiments, Dgrn suppressed the small eye phenotype associated with adults ectopically expressing Gro in the eye, a function that requires Dgrn’s catalytic activity and ability to bind SUMOylated proteins. Together, these findings established a role for Dgrn in antagonizing Hairy- and Gro-mediated repression in vivo [13].

Dgrn activity is not limited to Hairy and it also regulates other HES/E(spl) family proteins that play important roles in embryogenesis. Enhancer of split [E(spl)] proteins play pivotal roles in the specification and development of the central and peripheral nervous systems [47,48]. Overexpression of *E(spl)* genes results in a “bald phenotype”, in which E(spl)-expressing adults lack thoracic bristles. In agreement with Dgrn’s ability to bind E(spl) proteins via their basic domain, Dgrn co-expression suppresses the E(spl)-induced lack of bristle phenotype and restores bristle formation. Another process regulated by Dgrn is sex determination. In this case, Deadpan (Dpn), a HES-related family protein, acts as an autosomal counting protein that represses transcription of the master sex regulator gene, *Sex lethal (Sxl)* [49,50]. *Sxl* is expressed in females and is required for female development. In contrast, males neither require nor express *Sxl*, and its misexpression in males results in lethality [51]. Dgrn physically binds to Dpn, and Dgrn-null mutant embryos accordingly fail to express *Sxl*. Moreover, overexpression of Dgrn results in an ectopic expression of *Sxl* in males, which in turn leads to male lethality, suggesting that Dgrn limits Dpn activity during sex determination [16]. 

## 4. Dgrn Regulates Transcriptional Activation during Early Development and Adult Immune Response

Dgrn, and the activation of zygotic genes: The transcriptional activities of Dgrn and STUbL proteins are highly relevant also for transcriptional activation in both flies and vertebrates. The vertebrate ortholog of Dgrn, RNF4, was initially shown to function as a transcriptional co-activator that enhances androgen receptor-dependent gene activation [52]. In the fly and during early embryonic development, Dgrn is required for transcriptional activation of target genes of developmental pathways such as Wnt, Torso, Dpp, and Toll (e.g., *engrailed, tll, hkb, zen*, and *twist*) [16] [Orian and Kulton, personal communication]. Indeed, Dgrn plays a critical role in the transition from the expression of maternal genes to that of zygotic genes such as in the case of *zen* and *twist*. Twist is required for dorso-ventral patterning and *twist* mRNA transcription depends on the activity of the Toll pathway. Toll receptor activation initiates a signaling cascade leading to the nuclear translocation and activation of Dorsal, the NF-κB-related (REL) transcription factor, and of Dif, the Dorsal-related immunity factor [53,54,55,56,57].

The Toll pathway is required also later during larvae development and in the adult form for proper response to pathogens. Together with the immune deficiency pathway (Imd), these pathways make up the fly’s NF-κB-related network, which is essential for coping with bacteria, fungi, and viruses [57,58,59]. Not surprisingly, SUMOylation in the fly regulates innate immune response, the Toll pathway, and REL-transcription factors [60,61,62]. For example, Dorsal is SUMOylated on Lys 382, a SUMOylation that enhances the factor’s transcriptional activity. This SUMOylation site is highly conserved in the second *Drosophila* REL transcription factor Dif, as well as in the mammalian NF-κB factor p105, and glucocorticoid and androgen receptors [60]. It was therefore shown, using gain-of-function experiments in *Drosophila* S2 cells, that Dgrn expression, but not its catalytic inactive mutants, enhance NF-κB-dependent gene transcription. Moreover, Dgrn expression alleviates the inhibitory effects of the cytoplasmic NF-κB inhibitor Cactus (fly ortholog of IkBα), which is similar to that observed upon expression of SUMO E2, Ubc9 [19,63], and the repressive activity of Gro on NF-κB in reporter gene assays. This Dgrn potentiating activity in S2 cells was independent of Dif, the key REL/NF-kB transcription immune-related factor [19,56]. 

Dgrn also regulates innate responses in larvae and adult flies in vivo, and is required for Toll and IMD-dependent transcriptional activation upon infection: In the developing 3rd instar larvae, expression of Dgrn in immune cells resulted in the formation of Melanotic tumors [Orian and Kulton, personal communication]. These clusters of hyper-proliferative circulating hemocytes are involved in phagocytosis and immune signaling [59]. Melanotic tumors are observed in hyper-active Toll mutants and upon over-expression of Dorsal or Dif. They are also observed upon expression of activated mitogenic pathways such as RAS^V12^ and in loss-of-function mutant enzymes within the SUMO pathway [64]. The SUMOylation requirement for Dgrn-potentiating transcriptional activity, as well as the exact mechanisms involving Dgrn in the development of Melanotic tumors, are still, however, unknown. 

In the adult fly, Dgrn is essential for the systemic immune response to pathogenes; *dgrn*-null adult mutants are viable, yet females are sterile. Upon pathogenic challenges, however, these mutants rapidly succumb to infection, due probably to the inability of Dgrn mutants to express anti-microbial peptides (AMPs). AMPs are short peptides that are secreted from fat body cells in response to infection, and are mandatory for pathogen elimination [59]. *Dgrn*-mutant flies fail to express AMP genes downstream of both the IMD and Toll pathways, suggesting a general failure in the activation of immune-related genes [19]. While *dgrn* mutants failed to activate transcription of endogenous AMP genes, the expression of reporter transgenes of these AMPs, in the same animals, was strikingly indistinguishable from that of wild-type animals [Kulton and Orian, unpublished] [19]. This suggests that the in vivo substrate (or substrates) of Dgrn is not a protein within the signaling cascade or the basal transcriptional machinery, but rather is related to a chromatin function of Dgrn in the vicinity of the endogenous AMP genes. Indeed, and as discussed below, the association of RNF4 with nucleosomes is critical for RNF4 ability potentiate oncogenic transcription in human cancer cells [35].

## 5. Dgrn, the Local Regenerative Response, and Notch-Dependent Gene Expression

Further to its essential role in the systemic innate response to pathogens, Dgrn is also mandatory for the local response to infection in entry sites of pathogens like the gut epithelia [65]. The *Drosophila* adult midgut, which is highly similar to the vertebrate intestine, is a tissue characterized with rapid cell turnover that is able to regenerate upon tissue damage [66]. It is composed of four major types of epithelial cells: somatic intestinal stem cells (ISC) that either self-renew or mature into progenitor cells termed enteroblasts (EBs). EBs do not self-renew and differentiate either to mature polyploid enterocytes (ECs) or enteroendocrine hormone-producing cells (EE) [67]. In the gut epithelia and upon infection, a series of pathological events lead to the death of enterocytes. This results in a robust and rapid regenerative response of intestinal stem cells (ISCs), which is mediated, in part, by activation of the Notch pathway followed by the transcription of Notch-target genes in EBs [68,69]. 

In brief, Notch activation is initiated by its ligand Delta, which is expressed on the surface of ISCs, while the Notch receptor is expressed on the surface of the adjacent progenitors, EBs. The Delta~Notch interaction induces a sequence of proteolytic events leading to the cleavage and release of a Notch cytoplasmic tail, termed the Notch intracellular domain (N-ICD). Upon cleavage, N-ICD translocates to the nucleus, where it associates with the DNA binding factor CSL/RJkB to assemble a transcriptional activation complex that induces the expression of Notch target genes [66,70]. In wild-type flies and upon exposure of the gut epithelia to pathogenic microbes, a dramatic increase in Delta expression on the surface of ISC is noted. This is followed by activation of Notch pathway target genes in the neighboring EBs. In *dgrn* mutants, ISCs are unable to upregulate Delta expression in response to infection, and subsequently only minimal Notch-dependent transcriptional activation is observed in the adjacent EBs. This reduced activation is likely due not only to the lack of Delta, but is also related to a positive effect of the ligase on N-ICD stability and activity (see Section 6 below). Interestingly, Dgrn activity in the context of the local regenerative response in the gut is opposed by the ubiquitin-specific peptidase CG8334, whose vertebrate orthologs are USP11 and USP32. USP11 was recently found to be associated with RNF4 and to de-ubiquitinate heterotypic SUMO-ubiquitin chains [12], suggesting that the entire STUbL regulatory network is highly conserved from flies to humans. 

**Figure 3:**
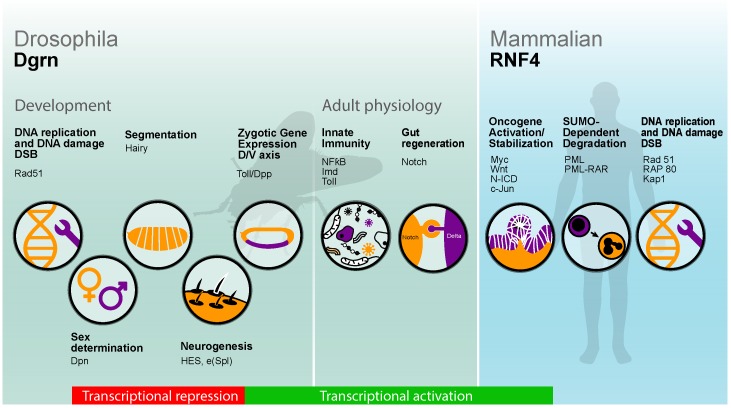
Processes and key proteins regulated by Dgrn and RNF4 in *Drosophila* and humans. In the developing embryo, Dgrn is required to resolve DSBs, and a similar function was attributed to RNF4 upon DNA damage in mouse and humans. In transcription, Dgrn determines co-factor choice during transcriptional repression limiting Hairy, Deadpan (Dpn), and HES E(spl) activity during segmentation, sex determination, and neurogenesis. Dgrn and RNF4 also enhance transcriptional activation. Dgrn is required for the expression of early zygotic genes such as *twist*, and *zen* downstream targets of the Toll and Dpp pathways. In the adult fly, Dgrn is required for the transcription of AMP genes, and for Notch-dependent transcription and gut regeneration. Likewise, RNF4 stabilizes and potentiates the transcriptional activity of c-Myc, c-Jun, and β-catenin promoting tumorigenesis of cancer cells. In contrast, in the context of promyelocytic leukemia, RNF4 ubiquitinates and targets the SUMOylated oncogenic PML-RAR for degradation, which suppresses tumorigenesis.

Thus, from the early stages of embryogenesis to adult life, Dgrn is intimately involved in transcriptional activation. The lessons gained from studies performed on the fly regarding the role of Dgrn in transcriptional activation are highly relevant to the function of mammalian STUbL proteins like RNF4 in transcriptional activation in the context of cancer, as outlined below (summarized in Figure 3).

## 6. RNF4 and Transcriptional Activation in Cancer

One prominent pathway co-discovered in *Drosophila* and mammary tumorigenesis is the Wnt pathway, which is essential for embryonic development and tumorigenesis [71]. In the absence of Wnt, β-catenin is either anchored to the cell membrane or degraded in the cytoplasm. Wnt/β-catenin target genes are repressed by the Gro/TLE co-repressor that associates with the DNA binding protein TCF/LEF and prevents activation of the Wnt pathway target genes. Upon Wnt pathway activation, β-catenin translocates to the nucleus, displacing Gro/TLE, and, together with TCF (*Drosophila* Pangolin), activates Wnt pathway target genes [72,73].

In the early embryo, Wnt/β-catenin (fly *Armadillo*) is required for the expression of *engrailed* and *dgrn*-hypomorphic embryos, which develop to this stage fail to properly express *engrailed* [16]. In human breast and colon cancer cells, the conditional loss of RNF4 likewise results in the inability to express *axin2*, a bona fide target of the Wnt pathway. Similar to Dgrn, RNF4 alleviates Gro/TLE-mediated repression of Wnt/β-catenin transcriptional activity, and RNF4 also potentiates the transcriptional activity of β-catenin, regardless of Gro/TLE. Moreover, and in line with that observed in the fly gut where Dgrn is required for Notch-dependent transcriptional activation, RNF4 potentiates Notch-dependent transcriptional activation in cancer cells. One common denominator of both Wnt and Notch pathways is the Myc oncogene, which is crucial to the tumorigenesis of these pathways [74,75,76]. Indeed, c-Myc binds to the genomic loci of *RNF4* and *RNF4* mRNA expression is elevated in Myc-driven cancers [77,78]. As described below, RNF4 stabilizes and potentiates c-Myc, Notch-Intercellular domain protein (N-ICD), and β-catenin, thus establishing a positive feed-forward loop and enhancing the activity of Wnt and Notch pathways. This potentiating activity is conserved in *Drosophila*. Expression of Dgrn in the ovaries restores the fertility of otherwise sterile hypomorphic Myc mutants that express low levels of dMyc (*dmyc^dm^*^1^) [79]. Dgrn expression was not, however, sufficient to rescue the lethality of *dmyc*-null mutants (*dmyc^dm^*^4^) [Orian, unpublished] [80], suggesting that RNF4 activity is aimed at, or requires, the c-Myc protein. 

Indeed, RNF4 is directly linked to transcriptional activation and tumorigenesis of selected oncoproteins including Myc [35]. While ubiquitination leads in many cases to proteasomal degradation, in the case of RNF4, ubiquitination of a subset of nuclear oncoproteins results in their stabilization and transcriptional hyper-activation rather than in their degradation. Among these phospho-oncoproteins are c-Myc, β-catenin, N-ICD, and c-Jun, all of which are required for G1/S transition and are rapidly degraded by SCF ubiquitin ligase complexes [45]. RNF4 specifically binds, ubiquitinates, and stabilizes these proteins, but only upon their phosphorylation by mitogenic kinases. As mentioned in Section 3, the conserved phosphorylation site (Ser62 within c-Myc) is part of a short stability box (“degron”) that is highly conserved also in several *Drosophila* transcription factors (Hb, Ftz, Prd, Eve). RNF4 recognition of these p-oncoproteins is mediated by a short arginine-rich region (ARM) that is also present in a modified version in Dgrn but not in RNF111, the second human STUbL. RNF4-dependent oncoprotein stabilization requires the catalysis of unique heterotypic poly-ubiquitin chains with internal linkages via K11 and K33 of ubiquitin, generating stabilized proteins that are hyper-active in transcription. 

It is possible that the transcriptional potentiating activities of Dgrn and RNF4 stem from distinct and different activities towards a variety of protein substrates. The general failure of rapid transcriptional activation in these diverse biological settings may, however, hint at a specific and shared role for Dgrn/RNF4. Indeed, all these RNF4-potentiating activities require association with chromatin. Interestingly, the requirement for Dgrn and RNF4 in activating transcription is similar to the activity of the transcription factor Zelda. Zelda is a Zinc-finger transcription factor that is required for genome activation that primes multiple loci for the activity of sequence transcription factors during maternal to zygotic transition [81,82]. It also shares similarity with the action of pioneer factors in mammalian cells [83].

One potentially shared molecular function in transcription that is attributed to RNF4 is a positive role in DNA demethylation, which is required for gene activation. Specifically, RNF4 was identified in a functional screen as a gene capable of activating otherwise methylated transcriptional reporter elements. Indeed, embryos lacking RNF4 are not viable and are characterized by increased genomic cytosine methylation [84]. It was suggested that the protein substrates of RNF4 in this context may be enzymes involved in DNA methylation, such as thymine-DNA glycosylase (TDG) [85] and MeCP2, a methyl-CpG-binding domain that is mutated in the female retardation Rett syndrome [86]. While RNF4 has been shown to be required for such de-methylation in human cells in vitro, the mechanisms involved in vivo are less clear, and RNF4/Dgrn-dependent chromatin-related mechanisms await further research. However, and regardless of the exact mechanisms involved, the transcriptional potentiating activity of RNF4 is crucial in the context of cancer. Expression of RNF4 in less aggressive colon and breast cancer cells (SW480 and MCF10, respectively) promotes tumor cell properties such as colony formation in soft agar. RNF4 is vital for the survival of aggressive colon and breast cancer cells, and high RNF4 protein levels are observed in biopsies derived from colon cancer patients during the transition from colon adenoma to carcinoma, and are correlated with poorer prognosis of luminal type-A breast cancer patients [35].

## 7. Concluding Remarks and Future Challenges

Studies from yeast, *Drosophila*, mice, and humans established critical roles for STUbL in diverse molecular and biological processes in development and cancer. As outlined above, both Dgrn and RNF4 have SUMO-dependent and independent modes of interaction with their substrates. A future challenge will be to identify the full spectrum of Dgrn/RNF4 substrates and to delineate the molecular rules that govern these different modes of recognition based on this large-scale analysis. 

A second unexplored area involves potential cytoplasmic functions of Dgrn and RNF4. Both proteins exhibit highly dynamic intracellular localization. In the fly embryo, the entire population of Dgrn protein alternates between the cytoplasm and nucleus during early embryonic cell cycles [16]. In human cells and in the cytoplasm, RNF4 was shown to be required for degradation of the cystic fibrosis transmembrane conductance regulator (CFTR) mutant F508del [87]. In patient-derived colon cancer biopsies, RNF4 was also localizes in the vicinity of the secretory pathway [35]. While we have so far addressed nuclear functions of Dgrn and RNF4, a future challenge will be to unveil cytoplasmic functions of STUbL, as well as the regulatory mechanism(s) that controls their intracellular localization. Studies in *Drosophila* will clearly be instrumental in addressing this question.

Importantly, all of the transcriptional and tumor-potentiating activities of RNF4 require both its ARM domain and binding to nucleosomes via a specific region at its C-terminus (NTR, Figure 1). Moreover, genetic ablation of RNF4 in aggressive breast cancer cells leads to their rapid death. Thus, while RNF4 itself is not an oncogene and is incapable of transforming primary cells, it fits well to an emerging group of proteins termed non-oncogene addiction genes (NOA) [88]. These genes are essential for the tumorous phenotype of cancer cells, but are less important to non-transformed cells. They are therefore likely to be important in early detection and may potentially serve as excellent molecular targets for the cure of cancer.
